# Sex hormone–binding globulin increases lumbar fracture risk via bone density, inflammation, and insulin pathways: A Mendelian randomization study

**DOI:** 10.1097/MD.0000000000050961

**Published:** 2026-10-02

**Authors:** Gushang Xia, Rong Yang, Yusong Zhou, Shenyu Luo, Jiannan Shao, Qingli Kong, Honggang Luo, Xianglin Li

**Affiliations:** a Department of Orthopaedics, The People’s Hospital of Chuxiong Yi Autonomous Prefecture, Chuxiong, China.

**Keywords:** bone mineral density, C-reactive protein, insulin medication, lumbar fracture, mediation analysis, Mendelian randomization, SHBG

## Abstract

Circulating sex hormone–binding globulin (SHBG) regulates bioavailable sex hormones that influence bone metabolism. We investigated whether genetically higher SHBG increases lumbar fracture (LF) risk and identified the biological pathways mediating this effect, including bone mineral density (BMD), C-reactive protein (CRP), and insulin use. A two-sample bidirectional and mediation Mendelian randomization (MR) framework was applied using genome-wide significant single-nucleotide polymorphisms (*P* < 5 × 10^−8^) from large-scale genome-wide association study datasets (FinnGen, UK Biobank, and IEU OpenGWAS). Instrumental variables were linkage disequilibrium-pruned (*r*^2^ < 0.01), and analyses were conducted using inverse-variance weighted, MR-Egger, and Mendelian Randomization Pleiotropy RESidual Sum and Outlier methods. Genetically predicted SHBG was causally associated with higher LF risk (FinnGen: odds ratio [OR] = 1.201; 95% confidence interval, 1.028–1.404; *P* = .021; UK Biobank: OR = 1.005; 95% confidence interval, 1.002–1.008; *P* = .002). SHBG showed significant negative effects on lumbar BMD (OR = 0.934; *P* = .0029) and CRP (OR = 0.921; *P* = .0065), and a modest decrease in insulin medication use (OR = 0.9963; *P* = 6.1 × 10^−6^). Mediation analysis revealed that 64.8% of SHBG’s total effect on LF was mediated through BMD, 63.8% through CRP, and 38.7% through insulin pathways. Sensitivity tests confirmed robustness without evidence of pleiotropy. Genetically elevated SHBG increases LF risk mainly through impaired bone density and systemic inflammation. These findings suggest that interventions targeting SHBG-related metabolic pathways may help reduce skeletal fragility.

## 1. Introduction

Lumbar fracture (LF) is a leading contributor to global musculoskeletal morbidity,^[1]^ occurring even among individuals whose bone mineral density (BMD) remains above clinical osteoporosis thresholds,^[2]^ thereby implicating additional physiological pathways in skeletal fragility.^[3,4]^ Sex hormone–binding globulin (SHBG) plays a central role in regulating the bioavailability of testosterone and estradiol,^[5,6]^ hormones that orchestrate bone remodeling via osteoblast and osteoclast activity, and modulate muscle mass,^[7–9]^ a key source of mechanical loading on bone. Elevated SHBG levels diminish free sex steroid levels, potentially disrupting bone resorption and impairing microarchitecture.^[10]^

Beyond bone mass, a constellation of intermediary processes – including systemic inflammation, glycemic control, and endocrine feedback loops – may mediate the influence of SHBG on fracture risk.^[4,11]^ C-reactive protein (CRP), a sensitive marker of inflammation, activates osteoclastogenesis and inhibits osteoblast differentiation.^[12–14]^ Insulin and glucose homeostasis intersect with bone metabolism through insulin receptor signaling in osteoblasts, while growth hormones (GHs) and bioavailable testosterone exert anabolic effects on bone and muscle.^[15–17]^ Vitamin D status further modulates calcium absorption and paracrine signaling within bone tissue.^[18,19]^

Establishing causality and quantifying mediation across these pathways poses challenges for traditional observational research due to confounding and reverse causation. Mendelian randomization (MR) leverages robust genetic proxies for exposure to infer directional effects, mitigating these biases.^[20–22]^ Two-sample MR extends this framework using summary statistics from large-scale genome-wide association studies (GWASs),^[23,24]^ providing statistical power to dissect complex trait interrelationships. Recent methodological advances, such as two-step and multivariable MR, enable the decomposition of total exposure-outcome effects into direct and indirect (mediated) components, illuminating the relative contributions of distinct biological pathways.^[24]^

In the present study, we applied a comprehensive MR approach to test whether genetically elevated SHBG increases the risk of lumbar vertebral fracture and parse the mechanisms by which this effect is transmitted. We analyzed SHBG’s causal impact^[25]^ on a broad panel of candidate mediators, including lumbar spine BMD (both overall and across age strata to capture developmental vs degenerative influences), CRP, insulin medication use, GH, bioavailable testosterone,^[26,27]^ glycated hemoglobin (HbA1c),^[28,29]^ and 25-hydroxyvitamin D (25(OH)D), and subsequently estimated each mediator’s effect on fracture risk.^[19,30,31]^ By integrating bidirectional, two-step, and multivariable MR analyses, we sought to delineate the endocrine, inflammatory, metabolic, and nutritional pathways that collectively govern vertebral bone integrity, thereby identifying priority targets for preventive intervention.^[32–34]^

## 2. Materials and methods

### 2.1. Study design

This study utilized a bidirectional, two-sample, and two-step MR approach to investigate the causal link between circulating SHBG and lumbar vertebral fracture and to uncover the mediating biological pathways. First, we conducted bidirectional MR analyses to assess whether genetically increased SHBG predisposes individuals to LF and, conversely, whether genetic liability to LF influences SHBG concentrations, thereby ruling out reverse causality. Next, we implemented a two-step MR framework in which SHBG’s causal effects on a panel of candidate mediators, including lumbar spine BMD, systemic inflammation (measured by C-reactive protein), insulin medication use, GH, bioavailable testosterone, HbA1c, and 25(OH)D – were estimated, followed by separate MR analyses of each mediator on LF risk. Finally, we applied multivariable inverse-variance weighted (IVW) MR together with a difference-of-coefficients method to decompose the overall SHBG–LF association into direct and indirect effects specific to each mediator, allowing quantification of the proportion of fracture risk transmitted through each pathway.

All genetic instruments were selected for genome-wide significance (*P* < 5 × 10^−8^), pruned to ensure independence (*r*^2^ < 0.01), and validated to have no associations with measured confounders^[35–37]^; we further verified the absence of horizontal pleiotropy using MR-Egger intercepts and Mendelian Randomization Pleiotropy RESidual Sum and Outlier (MR-PRESSO) tests. As all summary statistics were obtained from publicly accessible GWASs of European-ancestry participants,^[38]^ no additional ethical approval was required. The overall study design and analytical steps are illustrated in Figure 1.

**Figure 1.**
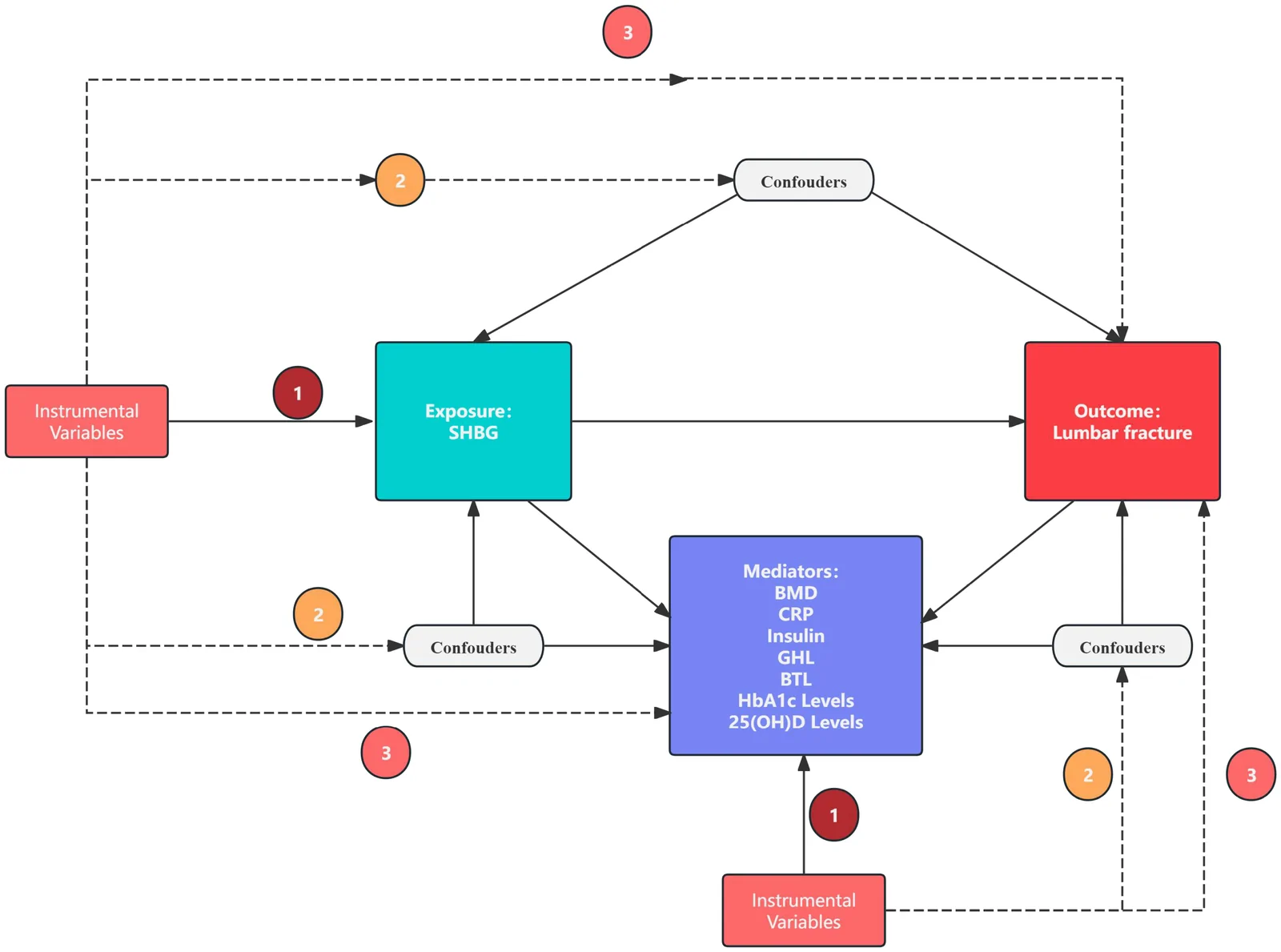
Study flow chart. ① IVs must be strongly associated with the exposures (SHBG and candidate mediators). ② IVs should not be associated with confounders of the exposure–outcome or mediator–outcome relationships. ③ IVs influence the outcome (LF) only through the exposures and mediators. Solid arrows denote validated causal or correlational paths, whereas dashed lines indicate the absence of correlation. 25(OH)D = 25-hydroxyvitamin D, BMD = total body bone mineral density, BTL = bioavailable testosterone levels, CRP = C-reactive protein, GHL = growth hormone levels, HbA1c = glycated hemoglobin, IV = instrumental variable, LF = lumbar fracture, SHBG = sex hormone–binding globulin.

Recognizing the well-documented lifecycle pattern of BMD – rapid accrual in childhood and adolescence, a plateau around age 30, and progressive decline after age 50, with accelerated loss postmenopause – we performed age-stratified MR of SHBG on BMD across 5 defined periods (0–15, 15–30, 30–45, 45–60, and >60 years) using a total body BMD dataset of 66,628 individuals (genetic factors for osteoporosis [GEFOS]/UK Biobank [UKB]) and site-specific measures at the femoral neck, lumbar spine, and forearm.

To further explore the potential biochemical mediators of skeletal effects of SHBG, we selected total body BMD, serum 25(OH)D, calcium supplementation, and SHBG itself, each supported by prior epidemiological evidence, to be tested in mediation analyses (see Tables S1, S2, S3, S4, S5, S6, S7, S8, S9, S10, S11, S12, S13, S14, S15, S16, S17, S18, S19, S20, S21, S22, S23, S24, S25, S26 and S27, Supplemental Digital Content 1, for dataset details). This comprehensive framework ensures rigorous causal inference and illuminates the specific biological processes through which SHBG impacts vertebral fracture risk.

### 2.2. Data sources

We derived genetic instruments and outcome data exclusively from large-scale, publicly available GWAS meta-analyses of individuals of European ancestry to minimize confounding by population stratification. SHBG instruments were obtained from the ebi-a-GCST90014011 meta-analysis (n SNPs = 363–418). LF summary statistics were drawn from FinnGen Release R7 (finn-b-ST19_FRACT_LUMBAR_SPINE_PELVIS; n SNPs = 12 for reverse MR) and UKB (ukb-b-15445; n SNPs = 18 for reverse MR). Mediator GWAS included lumbar spine BMD (GEFOS/UKB; ebi-a-GCST005348) with age-stratified subsets (0–15 years: GCST005345; 15–30 years: GCST005344; 30–45 years: GCST005346; 45–60 years: GCST005350; >60 years: GCST005349), CRP (ieu-b-4764), insulin medication use (ukb-b-15445), GH levels (ebi-a-GCST90012032), bioavailable testosterone (ebi-a-GCST90012104), HbA1c (ebi-a-GCST90014006), and serum 25(OH)D (ebi-a-GCST90000618). All GWAS provided effect estimates on the log odds scale for binary traits or standardized units for quantitative traits. Detailed information on GWAS datasets, sample sizes, and variable definitions is provided in Table S1, Supplemental Digital Content 1. Single-nucleotide polymorphism (SNP)-level instrumental variables (IVs) for the SHBG–LF analyses are provided in Tables S2 and S3, Supplemental Digital Content 2. IVs for SHBG–candidate mediator analyses are provided in Tables S4, S5, S6, S7, S8, S9, S10, S11, S12, S13, S14 and S15, Supplemental Digital Content 3. IVs for candidate mediator–LF analyses are provided in Tables S16, S17, S18, S19, S20, S21, S22, S23, S24, S25, S26 and S27, Supplemental Digital Content 4.

### 2.3. Selection of genetic instrumental variables

For each exposure and mediator, we selected independent SNPs meeting genome-wide significance (*P* < 5 × 10^−8^) and pruned for linkage disequilibrium at *r*^2^ < 0.01 within a 10 Mb window using the 1000 Genomes European reference panel. We harmonized the effect alleles across datasets, discarding palindromic variants with ambiguous minor allele frequencies. Instrument strength was quantified using the *F*-statistic (*F* > 10 threshold), and weak instruments were excluded. For multivariable MR, we constructed joint instruments by combining SNP sets and adjusting for pairwise linkage disequilibrium.^[39–42]^

### 2.4. Ethical approval

This study was based entirely on publicly available summary-level GWAS data. Ethical approval and informed consent were obtained in the original studies. No individual-level data were used in the present study; therefore, additional ethical approval and informed consent were not required.

### 2.5. Statistical analysis

Causal estimates were derived via IVW MR under a multiplicative random effects model to account for between-variant heterogeneity. We conducted complementary sensitivity analyses using the MR-Egger regression (to test and adjust for directional pleiotropy via the intercept term), weighted median estimation (robust if up to 50% of instruments are invalid), and simple/weighted mode approaches. Bidirectional MR applies the same methods in reverse. Age-stratified MR for BMD → LF was performed for each predefined age group.

Mediation effects were quantified in 2 ways: multivariable IVW MR, modeling SHBG and each mediator simultaneously to estimate direct effects (β_direct); and difference-of-coefficients, computing the indirect effect as β_total (univariable IVW) minus β_direct, with standard errors estimated via the delta method. The proportion mediated was calculated as β_indirect/β_total.

We assessed instrument heterogeneity using Cochran’s *Q* statistic for both the IVW and MR-Egger models. Directional pleiotropy was evaluated by testing the MR-Egger intercept against 0. MR-PRESSO was employed to detect and correct outlier SNPs using global testing. All analyses were performed in R (v4.2.2; R Core Team, R Foundation for Statistical Computing) using the TwoSampleMR (v0.5.6; Gibran Hemani, MRC Integrative Epidemiology Unit [MRC IEU], University of Bristol) and MRPRESSO packages (Marie Verbanck, Ron Do Lab, Icahn School of Medicine), with a two-sided *P* < .05 denoting statistical significance.^[43,44]^

## 3. Results

### 3.1. Effect of SHBG on lumbar vertebral fracture risk

IVW MR in FinnGen (n SNP = 376) demonstrated that each 1–standard-deviation increase in genetically predicted SHBG increased lumbar vertebral fracture odds by 20.1% (odds ratio [OR] = 1.201; 95% confidence interval [CI], 1.028–1.404; *P* = .021), a finding that was recapitulated in the UKB (n SNP = 369; OR = 1.005; 95% CI, 1.002–1.008; *P* = .002). Both weighted median and mode estimators yielded consistent effect directions, and nonsignificant MR-Egger intercepts (FinnGen *P* = .060; UKB *P* = .512) argued against horizontal pleiotropy. Reverse MR using fracture-associated SNPs (12 in FinnGen, 18 in UKB) showed no influence of LF liability on SHBG levels (all *P* > .3), confirming a unidirectional causal link between SHBG and fracture, as shown in Figure 2.

**Figure 2.**
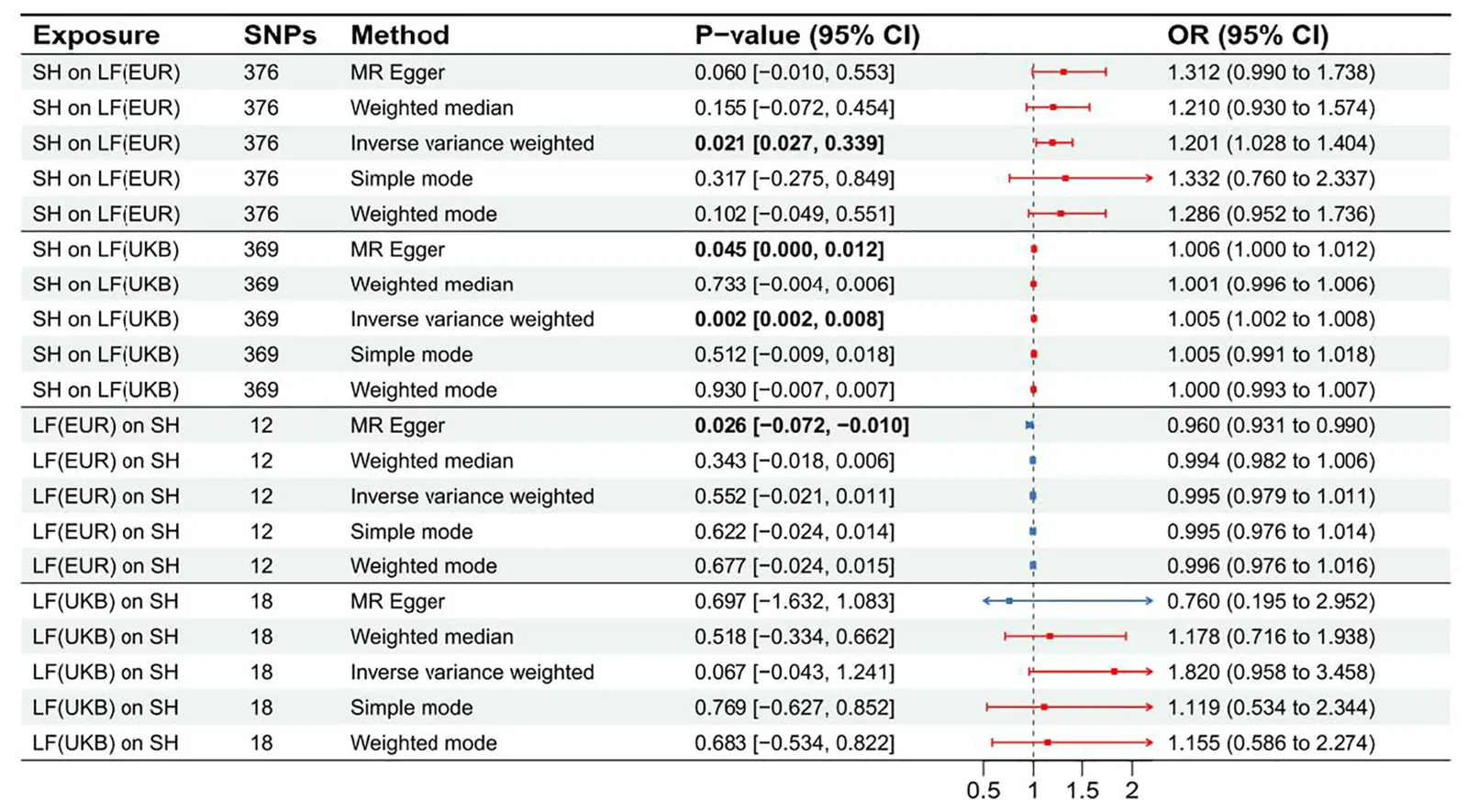
Mendelian randomization estimates for the causal effect of SHBG on lumbar vertebral fracture risk. Forest plot of ORs and 95% CIs for a 1–standard-deviation increase in genetically predicted SHBG on fracture risk in FinnGen and UK Biobank, using IVW, MR-Egger, weighted median, simple mode, and weighted mode methods. CI = confidence interval, IVW = inverse-variance weighted, LF = lumbar fracture, MR = Mendelian randomization, OR = odds ratio, SHBG = sex hormone–binding globulin, SNP = single-nucleotide polymorphism, UKB = UK Biobank.

### 3.2. Age-dependent impact of SHBG on lumbar spine BMD

By applying 415 SHBG instruments, IVW MR estimated a 6.6% overall decrease in lumbar spine BMD per standard-deviation increase in SHBG (OR = 0.934; 95% CI, 0.892–0.977; *P* = .0029). Stratified analyses revealed no significant associations during bone accrual and peak mass phases (0–15 years: OR = 1.025; *P* = .567; 15–30 years: OR = 0.901; *P* = .104; 30–45 years: OR = 0.971; *P* = .504), whereas inverse effects became significant in midlife (45–60 years: OR = 0.911; 95% CI, 0.849–0.978; *P* = .0099) and older adulthood (>60 years: OR = 0.874; 95% CI, 0.818–0.934; *P* = 6.7 × 10^−5^). These results indicate that SHBG’s deleterious influence on bone density emerges after attainment of peak BMD (Fig. 3).

**Figure 3.**
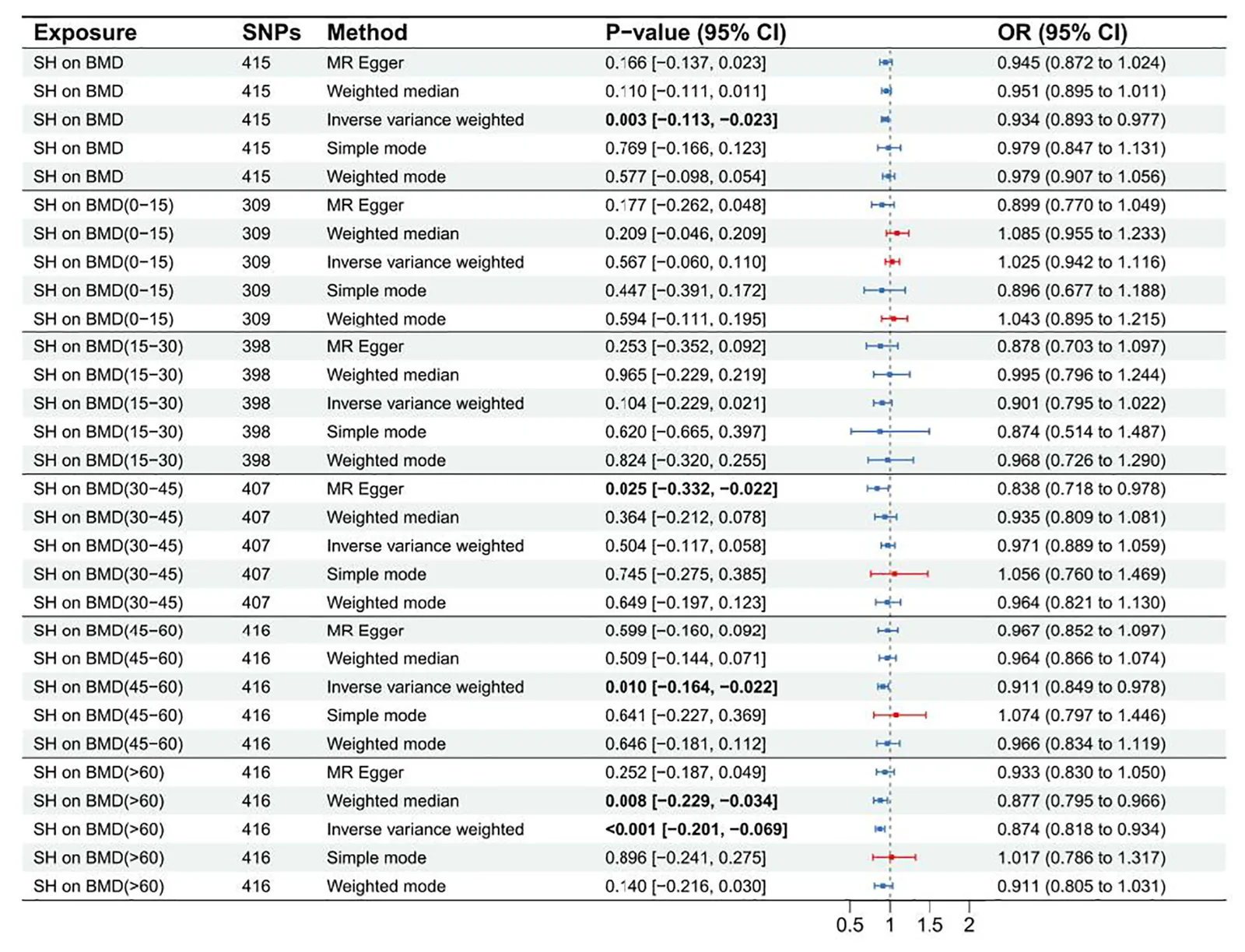
Mendelian randomization estimates of the causal effect of SHBG on lumbar spine BMD, overall and by age strata (0–15, 15–30, 30–45, 45–60, >60 years). Forest plot displays ORs and 95% CIs for a 1–standard-deviation increase in genetically predicted SHBG, as estimated by IVW (primary) alongside MR-Egger, weighted median, simple mode, and weighted mode methods. Significant negative associations are observed in the 45- to 60- and >60-year groups, indicating that SHBG-mediated reductions in BMD emerge in midlife and intensify in older age. BMD = bone mineral density, CI = confidence interval, IVW = inverse-variance weighted, OR = odds ratio, SHBG = sex hormone–binding globulin, SNP = single-nucleotide polymorphism.

### 3.3. Protective role of lumbar spine BMD against fracture

Using 126 independent BMD SNPs, IVW MR found that each standard deviation increase in lumbar spine BMD conferred a 32.7% reduction in vertebral fracture risk (OR = 0.673; 95% CI, 0.582–0.779; *P* = 1.04 × 10^−7^). Age-stratified estimates highlighted maximal protection in early life (0–15 years: OR = 0.801; 95% CI, 0.678–0.946; *P* = .0091) and late life (>60 years: OR = 0.808; 95% CI, 0.714–0.914; *P* = 6.66 × 10^−4^), with intermediate yet significant effects in midlife (45–60 years: OR = 0.920; 95% CI, 0.848–0.997; *P* = .0424; Fig. 4).

**Figure 4.**
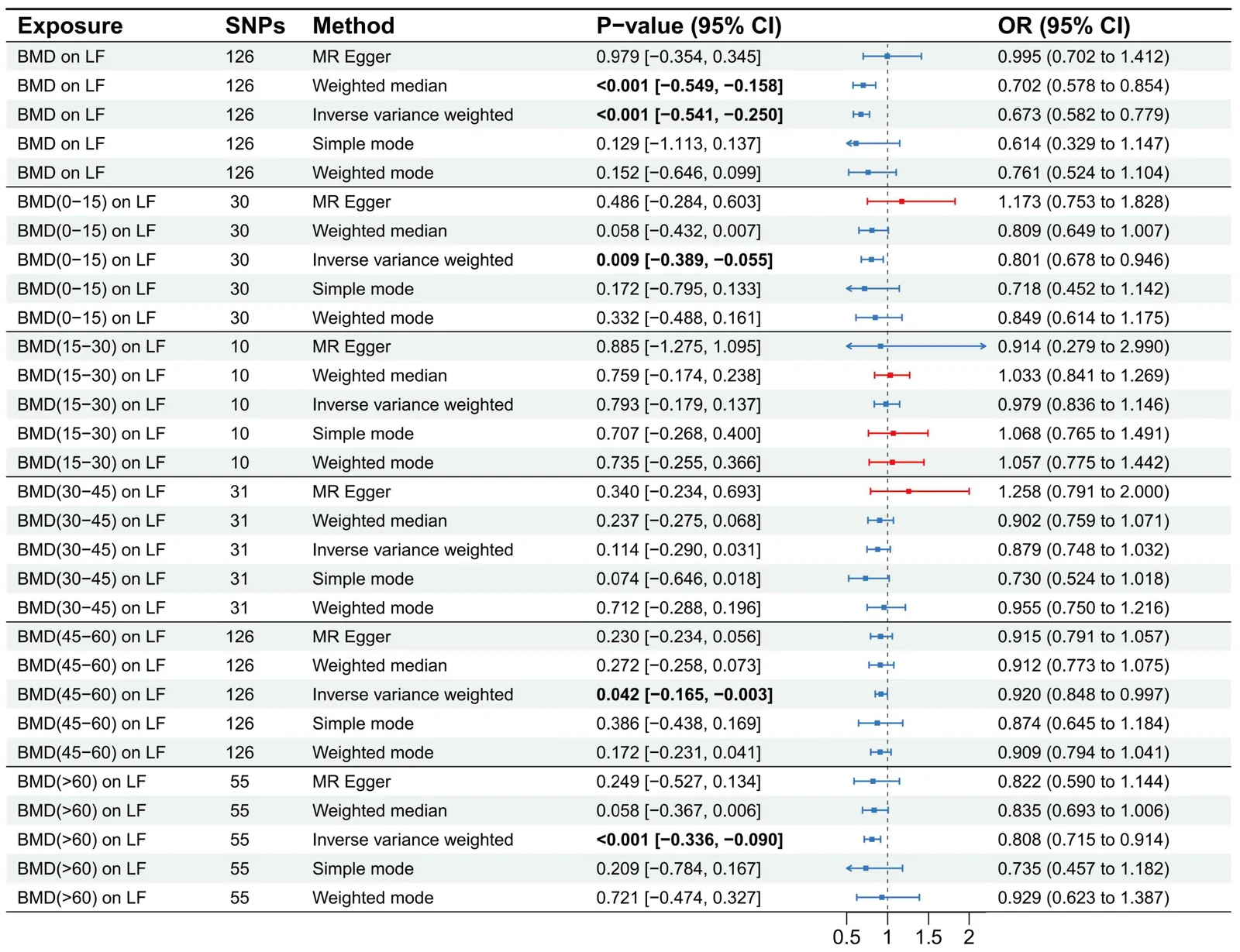
Causal effect estimates of lumbar spine BMD on lumbar vertebral fracture risk across the lifespan. Forest plot of inverse-variance weighted ORs and 95% CIs for a 1–standard-deviation increase in BMD overall and by age strata (0–15, 15–30, 30–45, 45–60, >60 years). Significant protective effects are observed overall (OR = 0.673; 95% CI, 0.582–0.779; *P* = 1.04 × 10^−7^) and in the 0- to 15-, 45- to 60-, and >60-year groups, with nonsignificant trends in the 15- to 30- and 30- to 45-year strata. BMD = bone mineral density, CI = confidence interval, LF = lumbar fracture, OR = odds ratio, SNP = single-nucleotide polymorphism.

### 3.4. Causal effects of SHBG on endocrine, inflammatory, and metabolic biomarkers

IVW MR analyses (n SNPs = 363–418) showed that higher SHBG significantly reduced whole-body BMD (OR = 0.934; *P* = .0029), lowered CRP (OR = 0.921; *P* = .0065), and modestly decreased insulin medication use (OR = 0.9963; *P* = 6.14 × 10^−6^). SHBG also increased GH levels (OR = 1.081; *P* = .0133), sharply suppressed bioavailable testosterone (OR = 0.758; *P* < 1 × 10^−145^), reduced HbA1c (OR = 0.859; *P* = 2.33 × 10^−13^), and elevated 25(OH)D (OR = 1.033; *P* = .0240), without evidence of pleiotropy (Fig. 5).

**Figure 5.**
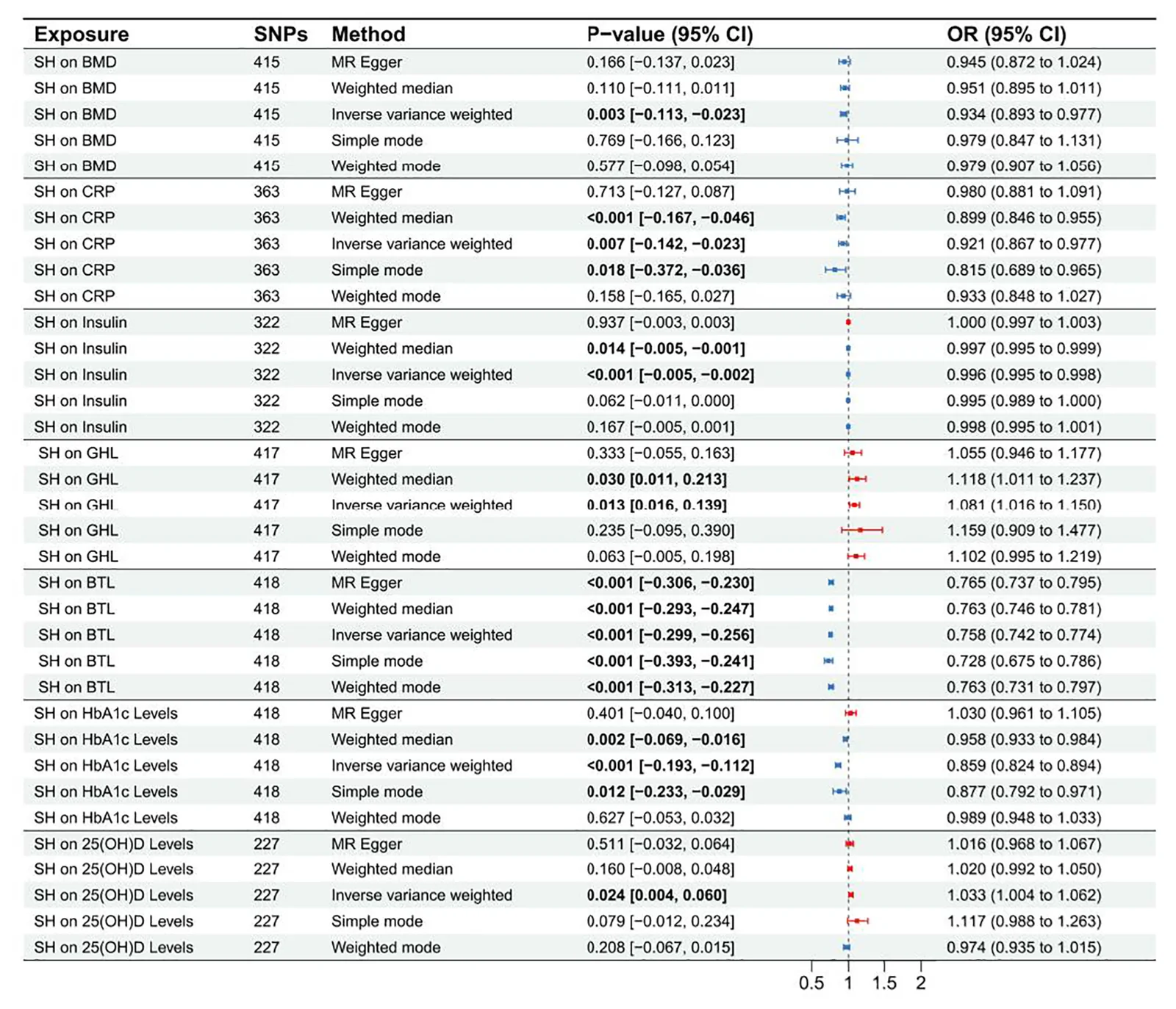
Causal effect estimates of SHBG on a panel of candidate mediators. Forest plot of inverse-variance weighted ORs and 95% CIs for a 1–standard-deviation increase in genetically predicted SHBG on: whole-body BMD (OR = 0.934; *P* = .0029), CRP (OR = 0.921; *P* = .0065), insulin medication use (OR = 0.9963; *P* = 6.1 × 10^−6^), growth hormone (OR = 1.081; *P* = .0133), bioavailable testosterone (OR = 0.758; *P* < 1 × 10^−145^), HbA1c (OR = 0.859; *P* = 2.33 × 10^−13^), and 25-hydroxyvitamin D (OR = 1.033; *P* = .0240). 25(OH)D = 25-hydroxyvitamin D, BMD = total body bone mineral density, BTL = bioavailable testosterone levels, CI = confidence interval, CRP = C-reactive protein, GHL = growth hormone levels, HbA1c = glycated hemoglobin, OR = odds ratio, SHBG = sex hormone–binding globulin, SNP = single-nucleotide polymorphism.

### 3.5. Mediator contributions to fracture risk

In reverse MR, increased whole-body and lumbar spine BMD again offered robust protection, whereas lower CRP was linked to a 14.4% reduction in fracture odds (OR = 0.856; 95% CI, 0.738–0.993; *P* = .039). Unexpectedly, higher insulin use was correlated with elevated fracture risk (OR = 22.3; *P* = .0499), likely reflecting the confounding effect of diabetes severity. GH, bioavailable testosterone, HbA1c, and 25(OH)D levels showed no significant effects (Fig. 6).

**Figure 6.**
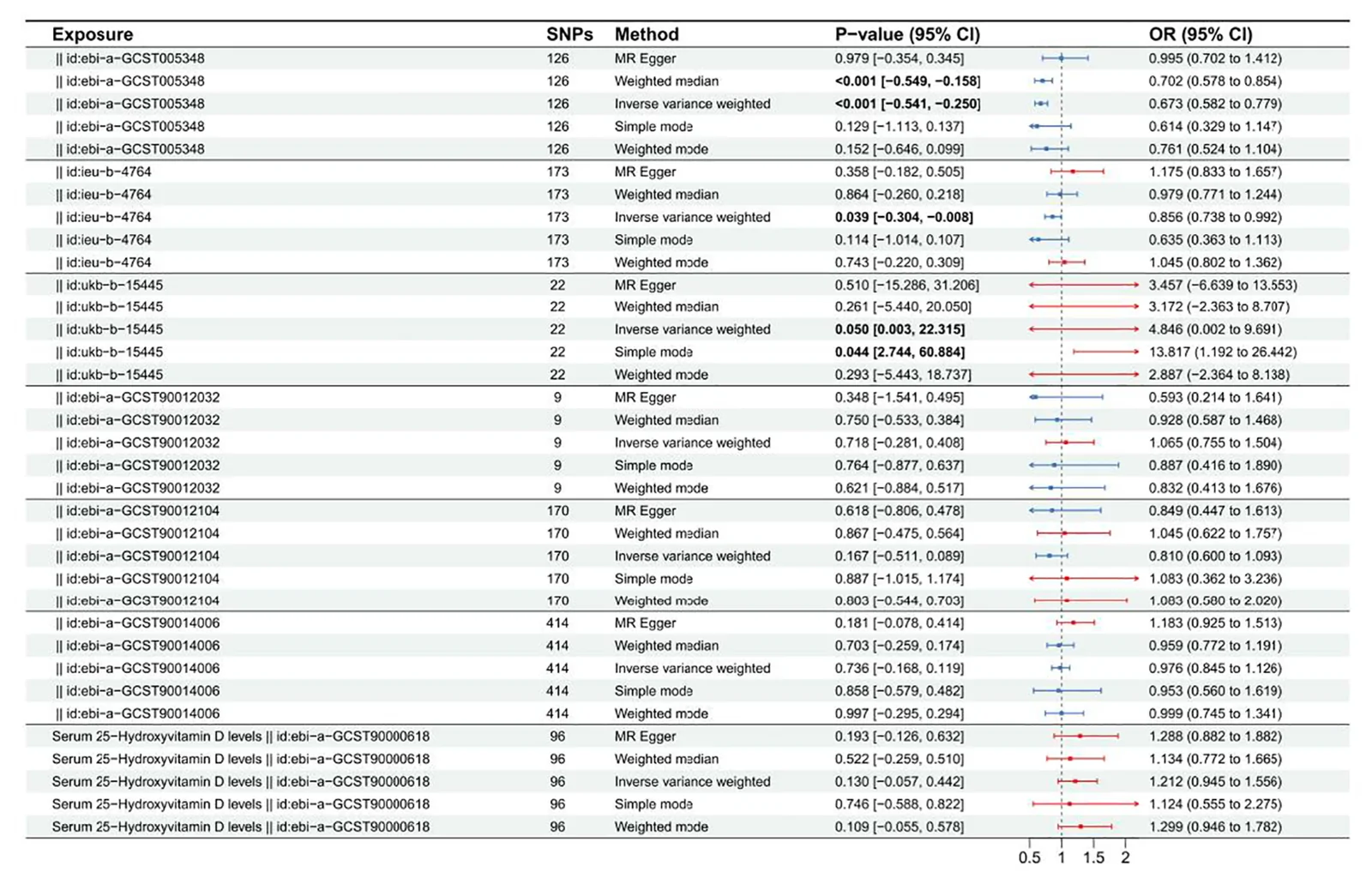
Causal effects of candidate mediators on lumbar vertebral fracture risk. Forest plot of inverse-variance weighted ORs and 95% CIs for a 1–standard-deviation increase in each mediator – lumbar spine BMD (overall and age-stratified: 0–15, 15–30, 30–45, 45–60, >60 years), whole-body BMD (GCST005348), CRP, insulin medication use, GH, bioavailable testosterone (Bio-T), HbA1c, and 25(OH)D – on vertebral fracture risk. Significant protective effects are observed for lumbar spine BMD overall (OR = 0.673; 95% CI, 0.582–0.779; *P* = 1.04 × 10^−7^) and in the 0- to 15-, 45- to 60-, and >60-year groups, as well as for lower CRP (OR = 0.856; 95% CI, 0.738–0.993; *P* = .039). Insulin use shows a positive association (OR ≈ 22.3; *P* = .0499), while other biomarkers exhibit nonsignificant effects. 25(OH)D = 25-hydroxyvitamin D, BMD = total body bone mineral density, CI = confidence interval, CRP = C-reactive protein, GH = growth hormone, HbA1c = glycated hemoglobin, OR = odds ratio, SNP = single-nucleotide polymorphism.

### 3.6. Quantification of mediation paths

Multivariable MR combined with the difference-of-coefficients approach revealed that 64.8% of SHBG’s total effect on LF (β_total = 0.1833) was mediated by BMD (β_indirect = 0.1187; SE = 0.112; OR_indirect = 1.126), 63.8% by CRP (β_indirect = 0.1170; SE = 0.113; OR_indirect = 1.124), and 38.7% by insulin use (β_indirect = 0.0710; SE = 0.122; OR_indirect = 1.074), underscoring bone density and inflammation as key conduits (Fig. 7).

**Figure 7.**
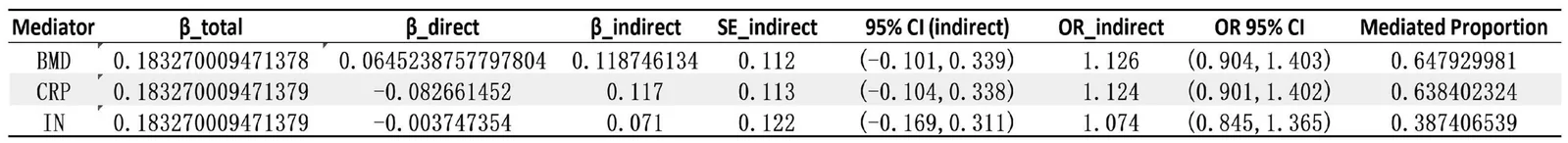
Mediation analysis of the SHBG-to-lumbar vertebral fracture pathway. Bar plot showing the proportion of the total effect (β_total) of SHBG on fracture mediated by lumbar spine BMD (64.8%), C-reactive protein (63.8%), and insulin medication use (38.7%). Indirect effect estimates (β_indirect) with standard errors and corresponding odds ratios (OR_indirect) are annotated for each mediator. BMD = total body bone mineral density, CI = confidence interval, CRP = C-reactive protein, IN = insulin, OR = odds ratio, SE = standard error, SHBG = sex hormone–binding globulin.

### 3.7. Robustness and sensitivity analyses

Cochran’s *Q* tests detected significant heterogeneity across most MR estimates (e.g., SHBG → BMD Q_IVW = 879.1; *P* < 1 × 10^−35^; SHBG → LF Q_IVW = 52.5; *P* = 2.2 × 10^−7^). MR-Egger intercepts were largely nonsignificant, indicating minimal directional pleiotropy, and MR-PRESSO identified outlier SNPs (all *P* < .001) whose removal did not materially alter the results, confirming the validity and robustness of our causal inferences (Fig. 8).

**Figure 8.**
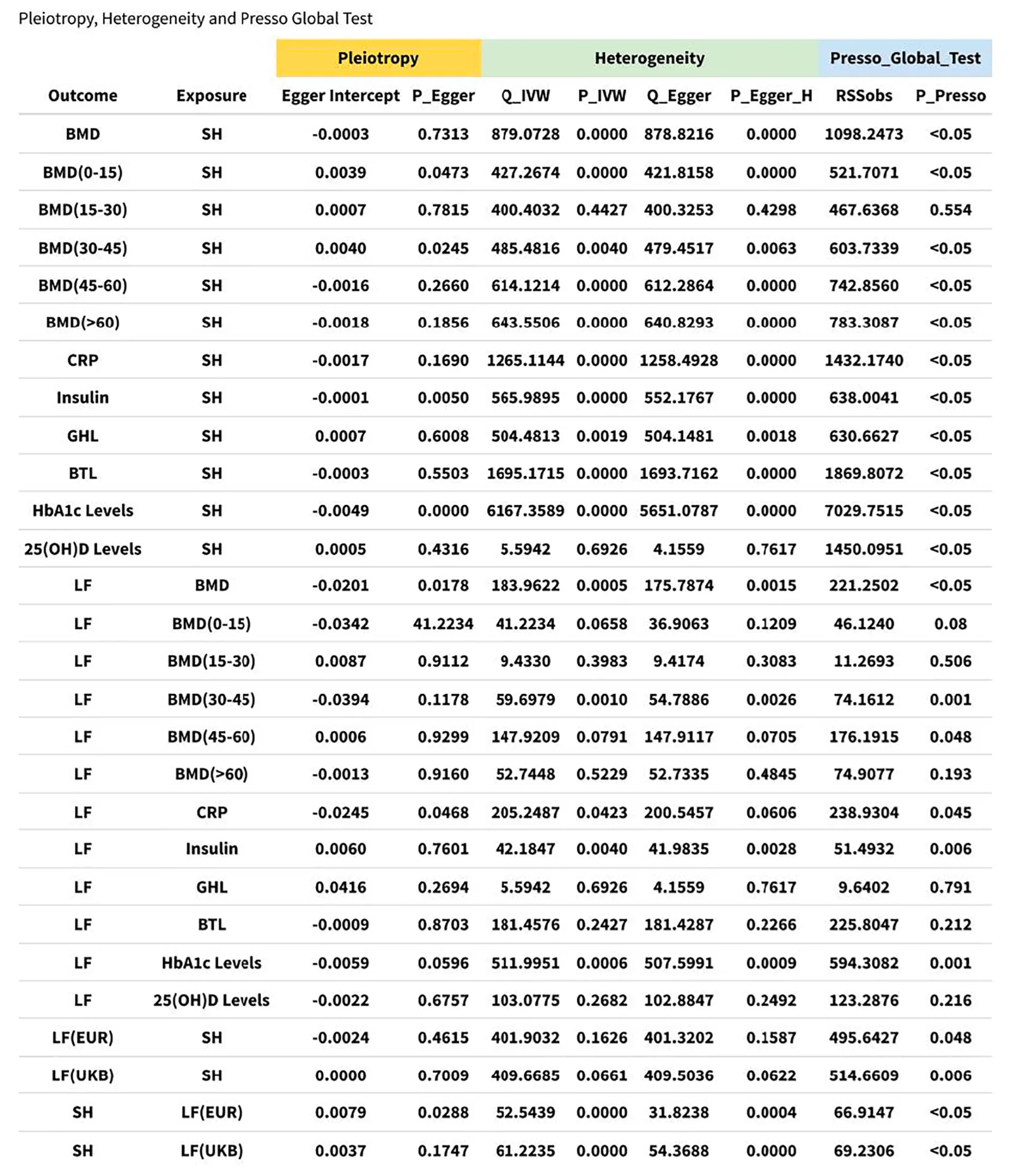
Assessment of heterogeneity and pleiotropy across Mendelian randomization analyses. Bar plot of Cochran’s *Q*–derived *P* values for IVW heterogeneity alongside MR-Egger intercept *P* values and MR-PRESSO global test *P* values for each exposure–outcome pair. Significant heterogeneity (Q_IVW *P* < .05) is observed for most SHBG → mediator and mediator → fracture paths, while nonsignificant MR-Egger intercepts indicate minimal directional pleiotropy. MR-PRESSO global tests detect outlier SNPs (*P* < .05), but correction does not alter causal estimates. 25(OH)D = 25-hydroxyvitamin D, BMD = total body bone mineral density, BTL = bioavailable testosterone levels, CRP = C-reactive protein, EUR = European ancestry, GHL = growth hormone levels, IVW = inverse-variance weighted, LF = lumbar fracture, MR-PRESSO = Mendelian Randomization Pleiotropy RESidual Sum and Outlier, SH = SHBG (sex hormone–binding globulin), SNP = single-nucleotide polymorphism, UKB = UK Biobank.

## 4. Discussion

In this comprehensive MR analysis, we provide compelling evidence that genetically elevated SHBG increases the risk of LF. Our bidirectional MR excluded reverse causation, and the IVW estimates were robust across sensitivity methods, indicating that the relationship is unlikely to be confounded or driven by pleiotropy. Importantly, we demonstrated that more than two-thirds of SHBG’s deleterious effect on fractures is mediated through reductions in BMD and modulation of systemic inflammation (CRP), with a smaller, yet biologically meaningful, contribution from altered insulin use patterns.^[45,46]^

Our age-stratified MR reveals that SHBG’s negative impact on lumbar spine BMD is negligible during bone accrual and maintenance phases (0–45 years) but becomes pronounced in midlife (45–60 years; OR = 0.911; *P* = .0099) and late adulthood (>60 years; OR = 0.874; *P* = 6.7 × 10^−5^). This age dependence aligns with the known trajectory of sex steroid decline and trabecular bone loss. During early life, abundant circulating estradiol and testosterone buffer the skeletal effects of SHBG variation, whereas after peak bone mass, even modest reductions in free sex steroids due to high SHBG disproportionately accelerate bone resorption and microarchitectural deterioration.^[47]^

The protective effect of lumbar spine BMD against fracture was strongest in the youngest (0–15 years) and oldest (>60 years) groups, highlighting the critical windows when bone mass has maximal influence on skeletal integrity. In youth, a higher BMD reflects optimal bone accrual, conferring lifelong mechanical advantage, whereas in the elderly, preserved BMD mitigates fracture risk despite age-related cortical thinning.

Our mediation analysis quantified that 64.8% of the total SHBG → fracture effect is transmitted via BMD, underscoring bone density as the principal conduit. SHBG’s potent suppression of bioavailable testosterone (OR = 0.758; *P* < 10^−145^) likely drives this pathway by impairing osteoblast differentiation and enhancing osteoclast activity. Concurrently, CRP levels mediate 63.8% of the effect; high SHBG reduces CRP (OR = 0.921; *P* = .0065), yet lower CRP itself protects against fracture (OR = 0.856; *P* = .039), suggesting that SHBG influences inflammatory signaling, perhaps via sex steroid–mediated modulation of immune cells, which in turn affects bone turnover.^[48,49]^

The 38.7% mediation by insulin use reflects SHBG’s role in metabolic regulation: high SHBG improves insulin sensitivity and reduces exogenous insulin requirements (OR = 0.9963; *P* = 6.14 × 10^−6^). Given the anabolic actions of insulin on the bone via osteoblast insulin receptors, reduced insulin exposure may contribute to diminished bone formation, although confounding by diabetes severity and medication type likely attenuates this estimate. Notably, other candidate mediators, such as GH, HbA1c, and 25(OH)D, showed no significant mediation, possibly because of smaller genetic effect sizes, compensatory endocrine mechanisms, or limited GWAS power to capture dynamic physiological fluctuations.^[50,51]^

This study has several limitations that merit consideration. Significant heterogeneity across MR analyses indicated variant-specific effects and underscored the complexity of SHBG’s biological actions. Although MR-Egger and MR-PRESSO did not detect systematic pleiotropy, the residual bias from unmeasured pathways could not be fully excluded. The indirect effect CIs overlapped the null, reflecting the imprecision inherent in mediation estimation; larger GWAS or individual-level data could enhance precision. Finally, our focus on European-ancestry populations limits extrapolation to other ethnic groups, warranting replication in diverse cohorts.^[52–54]^

Despite these caveats, our integrated MR approach delineates the hierarchical contributions of endocrine,^[55]^ skeletal, inflammatory, and metabolic pathways in SHBG-induced fracture risk. Therapeutically, interventions that lower SHBG levels or counteract its downstream effects, such as selective sex steroid supplementation, anti-inflammatory agents, or strategies to optimize bone density, may offer targeted means to reduce fracture incidence.^[49,56]^ Future research should explore the interplay between SHBG, sex steroids, and the local bone microenvironment, as well as evaluate potential gene–environment interactions that modulate these causal pathways.^[57]^

## 5. Conclusion

Our MR analyses established that elevated circulating SHBG causally increases lumbar vertebral fracture risk, with the majority of this effect mediated through reductions in BMD and modulation of systemic inflammation and a lesser contribution from altered insulin dynamics. Age-stratified evidence indicates that SHBG’s impact of SHBG on bone density intensifies after peak bone mass, while preserved BMD and controlled inflammation confer robust fracture protection across the life course. These findings delineate key mechanistic pathways – endocrine, skeletal, and inflammatory – linking SHBG to skeletal fragility and highlight BMD and CRP as priority targets for intervention. Therapeutic strategies aimed at modulating SHBG levels or mitigating their downstream effects on bone and inflammation may represent promising avenues for preventing vertebral fractures in at-risk populations.

## Acknowledgments

We gratefully acknowledge the investigators of the FinnGen Consortium, UK Biobank, GEFOS consortium, and IEU OpenGWAS project for publicly available GWAS summary statistics. We also thank all study participants and staff, whose efforts and contributions made this research possible.

## Author contributions

**Conceptualization:** Gushang Xia.

**Data curation:** Gushang Xia, Rong Yang, Yusong Zhou, Shenyu Luo, Jiannan Shao.

**Investigation:** Shenyu Luo, Jiannan Shao, Qingli Kong, Honggang Luo.

**Methodology:** Gushang Xia, Rong Yang, Honggang Luo.

**Supervision:** Xianglin Li.

**Validation:** Honggang Luo.

**Writing – original draft:** Gushang Xia, Xianglin Li.

**Writing – review & editing:** Gushang Xia, Xianglin Li.
























































