# Supplementary material for: Sex hormone–binding globulin increases lumbar fracture risk via bone density, inflammation, and insulin pathways: A Mendelian randomization study
Source: Medicine (Baltimore). 2026 Oct 2;105(40):e50961. doi: 10.1097/MD.0000000000050961 (PMC13633024; doi:10.1097/MD.0000000000050961)
Supplement: Supplementary file 1 [file medi-105-e50961-s028.docx]

**Sex Hormone–Binding Globulin Increases Lumbar Fracture Risk via Bone Density, Inflammation, and Insulin Pathways: A Mendelian Randomization Study**

**Gushang Xia,MD^1^,Rong Yang,MD^1^,Yusong Zhou,MMed^1^,Shenyu Luo,MMed^1^,Jiannan Shao,MMed^1^,Qingli Kong,MMed^1^,Honggang Luo,MMed^1^,Xianglin Li,PhD^1*^**

**^1^Department of Orthopaedics,The People's Hospital of Chuxiong Yi Autonomous Prefecture, Chuxiong, China**

***corresponding author information:**

**Xianglin Li.Department of Orthopaedics,The People's Hospital of Chuxiong Yi Autonomous Prefecture, Chuxiong, China**

**Telephone:（+86）0878-3164007**

**Email: [1171964433@qq.com](mailto:xiagushang5190@163.com)**

**[Abbreviations](mailto:xiagushang5190@163.com)**

[25(OH)D, 25-hydroxyvitamin D](mailto:xiagushang5190@163.com)

[BMD, bone mineral density](mailto:xiagushang5190@163.com)

[CI, confidence interval](mailto:xiagushang5190@163.com)

[CRP, C-reactive protein](mailto:xiagushang5190@163.com)

[GH, growth hormone](mailto:xiagushang5190@163.com)

[GWAS, genome-wide association study](mailto:xiagushang5190@163.com)

[HbA1c, glycated hemoglobin](mailto:xiagushang5190@163.com)

[IV, instrumental variable](mailto:xiagushang5190@163.com)

[IVW, inverse-variance weighted](mailto:xiagushang5190@163.com)

[LD, linkage disequilibrium](mailto:xiagushang5190@163.com)

[LF, lumbar fracture](mailto:xiagushang5190@163.com)

[MR, Mendelian randomization](mailto:xiagushang5190@163.com)

[MR-PRESSO, Mendelian Randomization Pleiotropy RESidual Sum and Outlier](mailto:xiagushang5190@163.com)

[OR, odds ratio](mailto:xiagushang5190@163.com)

[SD, standard deviation](mailto:xiagushang5190@163.com)

[SHBG, sex hormone-binding globulin](mailto:xiagushang5190@163.com)

[SNP, single-nucleotide polymorphism](mailto:xiagushang5190@163.com)

[UKB, UK Biobank](mailto:xiagushang5190@163.com)

**Abstract**

**Background:**

Circulating sex hormone–binding globulin (SHBG) regulates bioavailable sex hormones that influence bone metabolism. We investigated whether genetically higher SHBG increases lumbar vertebral fracture (LF) risk and identified the biological pathways mediating this effect, including bone mineral density (BMD), C-reactive protein (CRP), and insulin use.

**Methods:**

A two-sample bidirectional and mediation Mendelian randomization (MR) framework was applied using genome-wide significant SNPs (P < 5×10⁻⁸) from large-scale GWAS datasets (FinnGen, UK Biobank, and IEU OpenGWAS). Instrumental variables were LD-pruned (r² < 0.01), and analyses were conducted using inverse-variance weighted (IVW), MR-Egger, and MR-PRESSO methods.

**Results:**

Genetically predicted SHBG was causally associated with higher LF risk (FinnGen: OR = 1.201, 95% CI 1.028–1.404, P = 0.021; UK Biobank: OR = 1.005, 95% CI 1.002–1.008, P = 0.002). SHBG showed significant negative effects on lumbar BMD (OR = 0.934, P = 0.0029) and CRP (OR = 0.921, P = 0.0065), and a modest decrease in insulin medication use (OR = 0.9963, P = 6.1×10⁻⁶). Mediation analysis revealed that 64.8% of SHBG’s total effect on LF was mediated through BMD, 63.8% through CRP, and 38.7% through insulin pathways. Sensitivity tests confirmed robustness without evidence of pleiotropy.

**Conclusion:**

Genetically elevated SHBG increases lumbar fracture risk mainly through impaired bone density and systemic inflammation. These findings suggest that interventions targeting SHBG-related metabolic pathways may help reduce skeletal fragility.

**Keywords:**SHBG; lumbar fracture; Mendelian randomization; mediation analysis; bone mineral density; C-reactive protein; insulin medication.

**1. Introduction**

Lumbar fracture (LF) is a leading contributor to global musculoskeletal morbidity[1], occurring even among individuals whose bone mineral density (BMD) remains above clinical osteoporosis thresholds[2], thereby implicating additional physiological pathways in skeletal fragility[3, 4]. Sex hormone–binding globulin (SHBG) plays a central role in regulating the bioavailability of testosterone and estradiol[5, 6], hormones that orchestrate bone remodeling via osteoblast and osteoclast activity, and modulate muscle mass[7-9], a key source of mechanical loading on bone. Elevated SHBG levels diminish free sex steroid levels, potentially disrupting bone resorption and impairing microarchitecture[10].

Beyond bone mass, a constellation of intermediary processes—including systemic inflammation, glycemic control, and endocrine feedback loops—may mediate the influence of SHBG on fracture risk[11, 12]. C-reactive protein (CRP), a sensitive marker of inflammation, activates osteoclastogenesis and inhibits osteoblast differentiation[13-15]. Insulin and glucose homeostasis intersect with bone metabolism through insulin receptor signaling in osteoblasts, while growth hormones and bioavailable testosterone exert anabolic effects on bone and muscle[16-18]. Vitamin D status further modulates calcium absorption and paracrine signaling within bone tissue[19, 20].

Establishing causality and quantifying mediation across these pathways poses challenges for traditional observational research due to confounding and reverse causation. Mendelian randomization (MR) leverages robust genetic proxies for exposure to infer directional effects, mitigating these biases[21-23]. Two‐sample MR extends this framework using summary statistics from large-scale genome-wide association studies (GWAS)[24, 25], providing statistical power to dissect complex trait interrelationships. Recent methodological advances, such as two-step and multivariable MR, enable the decomposition of total exposure‐outcome effects into direct and indirect (mediated) components, illuminating the relative contributions of distinct biological pathways[26].

In the present study, we applied a comprehensive MR approach to test whether genetically elevated SHBG increases the risk of lumbar vertebral fracture and parses the mechanisms by which this effect is transmitted. We analyzed SHBG’s causal impact of SHBG [27]on a broad panel of candidate mediators, including lumbar spine BMD (both overall and across age strata to capture developmental versus degenerative influences), CRP, insulin medication use, growth hormone, bioavailable testosterone[28, 29], HbA1c[30, 31], and 25-hydroxyvitamin D, and subsequently estimated each mediator’s effect on fracture risk[32-34]. By integrating bidirectional, two-step, and multivariable MR analyses, we sought to delineate the endocrine, inflammatory, metabolic, and nutritional pathways that collectively govern vertebral bone integrity, thereby identifying priority targets for preventive intervention[35-37].

**2.Materials and methods**

**2.1 Study Design**

This study utilized a bidirectional, two‐sample, and two‐step Mendelian randomization (MR) approach to investigate the causal link between circulating sex hormone–binding globulin (SHBG) and lumbar vertebral fracture (LF) and to uncover the mediating biological pathways. First, we conducted bidirectional MR analyses to assess whether genetically increased SHBG predisposes individuals to LF and, conversely, whether genetic liability to LF influences SHBG concentrations, thereby ruling out reverse causality. Next, we implemented a two-step MR framework in which SHBG’s causal effects of SHBG on a panel of candidate mediators, including lumbar spine bone mineral density (BMD), systemic inflammation (measured by C‐reactive protein), insulin medication use, growth hormone, bioavailable testosterone, glycated hemoglobin (HbA1c), and 25‐hydroxyvitamin D—were estimated, followed by separate MR analyses of each mediator on LF risk. Finally, we applied multivariable inverse-variance weighted MR together with a difference‐of‐coefficients method to decompose the overall SHBG–LF association into direct and indirect effects specific to each mediator, allowing quantification of the proportion of fracture risk transmitted through each pathway.

All genetic instruments were selected for genome‐wide significance (P < 5×10⁻⁸), pruned to ensure independence (r² < 0.01), and validated to have no associations with measured confounders[38-40]; we further verified the absence of horizontal pleiotropy using MR‐Egger intercepts and MR‐PRESSO tests. As all summary statistics were obtained from publicly accessible GWAS of European‐ancestry participants[41], no additional ethical approval was required. The overall study design and analytical steps are illustrated in Figure 1.

Recognizing the well‐documented lifecycle pattern of BMD—rapid accrual in childhood and adolescence, a plateau around age 30, and progressive decline after age 50, with accelerated loss postmenopause —we performed age‐stratified MR of SHBG on BMD across five defined periods (0–15, 15–30, 30–45, 45–60, and >60 years) using a total body BMD dataset of 66,628 individuals (GEFOS/UKB) and site‐specific measures at the femoral neck, lumbar spine, and forearm.

To further explore the potential biochemical mediators of skeletal effects of SHBG, we selected total body BMD, serum 25‐hydroxyvitamin D, calcium supplementation, and SHBG itself, each supported by prior epidemiological evidence, to be tested in mediation analyses (see Supplementary Table S1 - S27 for dataset details). This comprehensive framework ensures rigorous causal inference and illuminates the specific biological processes through which SHBG impacts vertebral fracture risk.

**2.2 Data Sources**

We derived genetic instruments and outcome data exclusively from large-scale, publicly available GWAS meta-analyses of individuals of European ancestry to minimize confounding by population stratification. SHBG instruments were obtained from ebi‐a‐GCST90014011 meta‐analysis (n SNPs = 363–418). LF summary statistics were drawn from FinnGen Release R7 (finn‐b‐ST19_FRACT_LUMBAR_SPINE_PELVIS; n SNPs = 12 for reverse MR) and UK Biobank (ukb‐b‐15445; n SNPs = 18 for reverse MR). Mediator GWAS included lumbar spine BMD (GEFOS/UKB; ebi‐a‐GCST005348) with age-stratified subsets (0–15 years: GCST005345; 15–30 years: GCST005344; 30–45 years: GCST005346; 45–60 years: GCST005350; >60 years: GCST005349), CRP (ieu‐b‐4764), insulin medication use (ukb‐b‐15445), growth hormone levels (ebi‐a‐GCST90012032), bioavailable testosterone (ebi‐a‐GCST90012104), HbA1c (ebi‐a‐GCST90014006), and serum 25(OH)D (ebi‐a‐GCST90000618). All GWAS provided effect estimates on the log odds scale for binary traits or standardized units for quantitative traits.Detailed information on GWAS datasets, sample sizes, and variable definitions is provided in Supplementary Table S1. SNP-level instrumental variables for the SHBG–lumbar fracture analyses are provided in Supplementary Tables S2 and S3. Instrumental variables for SHBG–candidate mediator analyses are provided in Supplementary Tables S4, S5, S6, S7, S8, S9, S10, S11, S12, S13, S14, and S15. Instrumental variables for candidate mediator–lumbar fracture analyses are provided in Supplementary Tables S16, S17, S18, S19, S20, S21, S22, S23, S24, S25, S26, and S27.

**2.3 Selection of Genetic Instrumental Variables**

For each exposure and mediator, we selected independent SNPs meeting genome-wide significance (P < 5×10⁻⁸) and pruned for linkage disequilibrium (LD) at r² < 0.01 within a 10 Mb window using the 1000 Genomes European reference panel. We harmonized the effect alleles across datasets, discarding palindromic variants with ambiguous minor allele frequencies. Instrument strength was quantified using the F-statistic (F > 10 threshold), and weak instruments were excluded. For multivariable MR, we constructed joint instruments by combining SNP sets and adjusting for pairwise LD[42-45].

**2.4 Ethical approval**

This study was based entirely on publicly available summary-level genome-wide association study data. Ethical approval and informed consent had been obtained in the original studies. No individual-level data were used in the present study; therefore, additional ethical approval and informed consent were not required.

**2.5 Statistical Analysis**

Causal estimates were derived via inverse-variance weighted (IVW) MR under a multiplicative random effects model to account for between‐variant heterogeneity. We conducted complementary sensitivity analyses using the MR‐Egger regression (to test and adjust for directional pleiotropy via the intercept term), weighted median estimation (robust if up to 50% of instruments are invalid), and simple/weighted mode approaches. Bidirectional MR applies the same methods in reverse. Age‐stratified MR for BMD→LF was performed for each predefined age group.

Mediation effects were quantified in two ways: (1) Multivariable IVW MR, modeling SHBG and each mediator simultaneously to estimate direct effects (β_direct); and (2) difference-of-coefficients, computing the indirect effect as β_total (univariable IVW) minus β_direct, with standard errors estimated via the delta method. The proportion mediated was calculated asβ_indirect / β_total..

We assessed instrument heterogeneity using Cochran’s Q statistic for both the IVW and MR‐Egger models. Directional pleiotropy was evaluated by testing the MR‐Egger intercept against zero. MR‐PRESSO was employed to detect and correct outlier SNPs using global testing. All analyses were performed in R (v4.2.2) using the TwoSampleMR (v0.5.6) and MRPRESSO packages, with a two-sided P < 0.05 denoting statistical significance[46, 47].

3. **Results**
   3.1 **Effect of SHBG on Lumbar Vertebral Fracture Risk**

Inverse-variance weighted MR in FinnGen (n SNP = 376) demonstrated that each one‐standard‐deviation genetically predicted an increase in SHBG and increased lumbar vertebral fracture odds by 20.1% (OR = 1.201; 95% CI, 1.028–1.404; P = 0.021), a finding that was recapitulated in the UK Biobank (n SNP = 369; OR = 1.005; 95% CI, 1.002–1.008; P = 0.002). Both weighted median and mode estimators yielded consistent effect directions, and non-significant MR‐Egger intercepts (FinnGen P = 0.060; UKB P = 0.512) argued against horizontal pleiotropy. Reverse MR using fracture‐associated SNPs (12 in FinnGen, 18 in UKB) showed no influence of LF liability on SHBG levels (all P > 0.3), confirming a unidirectional causal link between SHBG and fracture, as shown in Figure 2.

3.2 **Age‐Dependent Impact of SHBG on Lumbar Spine BMD**

By applying 415 SHBG instruments, IVW MR estimated a 6.6% overall decrease in lumbar spine BMD per SD increase in SHBG (OR, 0.934; 95% CI, 0.892–0.977; P = 0.0029). Stratified analyses revealed no significant associations during bone accrual and peak mass phases (0–15 years: OR = 1.025, P = 0.567; 15–30 years: OR = 0.901, P = 0.104; 30–45 years: OR = 0.971, P = 0.504), whereas inverse effects became significant in midlife (45–60 years: OR = 0.911; 95% CI, 0.849–0.978; P = 0.0099) and older adulthood (>60 years: OR = 0.874; 95% CI, 0.818–0.934; P = 6.7×10⁻⁵). These results indicate that SHBG’s deleterious influence of SHBG on bone density emerges after attainment of peak BMD (Figure 3).

3.3 **Protective Role of Lumbar Spine BMD Against Fracture**

Using 126 independent BMD SNPs, IVW MR found that each SD increase in lumbar spine BMD conferred a 32.7% reduction in vertebral fracture risk (OR = 0.673; 95% CI, 0.582–0.779; P = 1.04×10⁻⁷). Age‐stratified estimates highlighted maximal protection in early life (0–15 years: OR = 0.801; 95% CI, 0.678–0.946; P = 0.0091) and late life (>60 years: OR = 0.808; 95% CI, 0.714–0.914; P = 6.66×10⁻⁴), with intermediate yet significant effect in midlife (45–60 years: OR = 0.920; 95% CI, 0.848–0.997; P = 0.0424) (Figure 4).

3.4 **Causal Effects of SHBG on Endocrine, Inflammatory, and Metabolic Biomarkers**

IVW MR analyses (n SNPs = 363–418) showed that higher SHBG significantly reduced whole‐body BMD (OR = 0.934; P = 0.0029), lowered CRP (OR = 0.921; P = 0.0065), and modestly decreased insulin medication use (OR = 0.9963; P = 6.14×10⁻⁶). SHBG also increased growth hormone levels (OR = 1.081; P = 0.0133), sharply suppressed bioavailable testosterone (OR = 0.758; P < 1×10⁻¹⁴⁵), reduced HbA1c (OR = 0.859; P = 2.33×10⁻¹³), and elevated 25‐hydroxyvitamin D (OR = 1.033; P = 0.0240), without evidence of pleiotropy (Figure 5).

3.5 **Mediator Contributions to Fracture Risk**

In reverse MR, increased whole‐body and lumbar spine BMD again offered robust protection, whereas lower CRP was linked to a 14.4% reduction in fracture odds (OR = 0.856; 95% CI, 0.738–0.993; P = 0.039). Unexpectedly, higher insulin use was correlated with elevated fracture risk (OR = 22.3; P = 0.0499), likely reflecting the confounding effect of diabetes severity. Growth hormone, bioavailable testosterone, HbA1c, and 25(OH)D levels showed no significant effects (Figure 6).

3.6 **Quantification of Mediation Paths**

Multivariable MR combined with the difference‐of‐coefficients approach revealed that 64.8% of SHBG’s total effect of SHBG on LF (β_total = 0.1833) was mediated by BMD (β_indirect = 0.1187; SE = 0.112; OR_indirect = 1.126), 63.8% by CRP (β_indirect = 0.1170; SE = 0.113; OR_indirect = 1.124), and 38.7% by insulin use (β_indirect = 0.0710; SE = 0.122; OR_indirect = 1.074), underscoring bone density and inflammation as key conduits (Figure 7).

3.7 **Robustness and Sensitivity Analyses**

Cochran’s Q tests detected significant heterogeneity across most MR estimates (e.g., SHBG→BMD Q_IVW = 879.1; P < 1×10⁻³⁵; SHBG→LF Q_IVW = 52.5; P = 2.2×10⁻⁷). MR‐Egger intercepts were largely non-significant, indicating minimal directional pleiotropy, and MR‐PRESSO identified outlier SNPs (all P < 0.001) whose removal did not materially alter the results, confirming the validity and robustness of our causal inferences (Figure 8).

4. **Discussion**

In this comprehensive Mendelian randomization (MR) analysis, we provide compelling evidence that genetically elevated sex hormone–binding globulin (SHBG) increases the risk of lumbar vertebral fracture (LF). Our bidirectional MR excluded reverse causation, and the inverse-variance weighted (IVW) estimates were robust across sensitivity methods, indicating that the relationship is unlikely to be confounded or driven by pleiotropy. Importantly, we demonstrated that more than two-thirds of SHBG’s deleterious effect on fractures is mediated through reductions in bone mineral density (BMD) and modulation of systemic inflammation (C-reactive protein; CRP), with a smaller, yet biologically meaningful, contribution from altered insulin use patterns[48, 49].

Our age-stratified MR reveals that SHBG’s negative impact on lumbar spine BMD is negligible during bone accrual and maintenance phases (0–45 years) but becomes pronounced in midlife (45–60 years; OR=0.911; P=0.0099) and late adulthood (>60 years; OR=0.874; P=6.7×10⁻⁵). This age dependence aligns with the known trajectory of sex steroid decline and trabecular bone loss. During early life, abundant circulating estradiol and testosterone buffer the skeletal effects of SHBG variation, whereas after peak bone mass, even modest reductions in free sex steroids due to high SHBG disproportionately accelerate bone resorption and microarchitectural deterioration[50].

The protective effect of lumbar spine BMD against fracture was strongest in the youngest (0–15 years) and oldest (>60 years) groups, highlighting the critical windows when bone mass has maximal influence on skeletal integrity. In youth, a higher BMD reflects optimal bone accrual, conferring lifelong mechanical advantage, whereas in the elderly, preserved BMD mitigates fracture risk despite age-related cortical thinning.

Our mediation analysis quantified that 64.8% of the total SHBG→fracture effect is transmitted via BMD, underscoring bone density as the principal conduit. SHBG’s potent suppression of bioavailable testosterone (OR=0.758; P<10⁻¹⁴⁵) likely drives this pathway by impairing osteoblast differentiation and enhancing osteoclast activity. Concurrently, CRP levels mediate 63.8% of the effect; high SHBG reduces CRP (OR=0.921; P=0.0065), yet lower CRP itself protects against fracture (OR=0.856; P=0.039), suggesting that SHBG influences inflammatory signaling, perhaps via sex steroid–mediated modulation of immune cells, which in turn affects bone turnover[51, 52].

The 38.7% mediation by insulin use reflects SHBG’s role in metabolic regulation: high SHBG improves insulin sensitivity and reduces exogenous insulin requirements (OR=0.9963; P=6.14×10⁻⁶). Given the anabolic actions of insulin on the bone via osteoblast insulin receptors, reduced insulin exposure may contribute to diminished bone formation, although confounding by diabetes severity and medication type likely attenuates this estimate. Notably, other candidate mediators, such as growth hormone, HbA1c, and 25-hydroxyvitamin D, showed no significant mediation, possibly because of smaller genetic effect sizes, compensatory endocrine mechanisms, or limited GWAS power to capture dynamic physiological fluctuations[53, 54].

This study has several limitations that merit consideration. Significant heterogeneity across MR analyses indicated variant-specific effects and underscored the complexity of SHBG’s biological actions of SHBG. Although MR-Egger and MR-PRESSO did not detect systematic pleiotropy, the residual bias from unmeasured pathways could not be fully excluded. The indirect effect confidence intervals overlapped the null, reflecting the imprecision inherent in mediation estimation; larger GWAS or individual-level data could enhance precision. Finally, our focus on European-ancestry populations limits extrapolation to other ethnic groups, warranting replication in diverse cohorts[55-57].

Despite these caveats, our integrated MR approach delineates the hierarchical contributions of endocrine[58], skeletal, inflammatory, and metabolic pathways in SHBG-induced fracture risk. Therapeutically, interventions that lower SHBG levels or counteract its downstream effects, such as selective sex steroid supplementation, anti-inflammatory agents, or strategies to optimize bone density, may offer targeted means to reduce fracture incidence[52, 59]. Future research should explore the interplay between SHBG, sex steroids, and the local bone microenvironment as well as evaluate potential gene–environment interactions that modulate these causal pathways[60].

5. **Conclusion**

Our Mendelian randomization analyses established that elevated circulating SHBG causally increases lumbar vertebral fracture risk, with the majority of this effect mediated through reductions in bone mineral density and modulation of systemic inflammation and a lesser contribution from altered insulin dynamics. Age‐stratified evidence indicates that SHBG’s impact of SHBG on bone density intensifies after peak bone mass, while preserved BMD and controlled inflammation confer robust fracture protection across the life course. These findings delineate key mechanistic pathways—endocrine, skeletal, and inflammatory—linking SHBG to skeletal fragility and highlight BMD and CRP as priority targets for intervention. Therapeutic strategies aimed at modulating SHBG levels or mitigating their downstream effects on bone and inflammation may represent promising avenues for preventing vertebral fractures in at-risk populations.

6. **Acknowledgments**

We gratefully acknowledge the investigators of the FinnGen Consortium, UK Biobank, GEFOS consortium, and IEU OpenGWAS project for publicly available GWAS summary statistics. We also thank all study participants and staff, whose efforts and contributions made this research possible.

**7.Funding Statement**

This research received no specific grants from any funding agency in the public, commercial, or not-for-profit sectors.

**8.Data availability statement**

The original contributions presented in this study are included in the article and Supplemental Digital Content. Further inquiries may be directed to the corresponding author.

9. **Author contributions**

Xia spearheaded the study as the first author: conceptualized the research framework, performed data curation, conducted the primary Mendelian randomization and mediation analyses, and drafted the manuscript. Rong Yang assisted with the methodological design and interpretation of genetic instruments. Yusong Zhou performed the sensitivity and pleiotropy assessments. Shenyu Luo performed age-stratified analyses and contributed to the data visualization. Shao managed GWAS summary statistics acquisition and harmonization. Qingli Kong supported the literature review and edited the manuscript. Honggang Luo provided statistical oversight and critical input for the analysis workflow. Xianglin Li supervised the project, guided the study design, and critically revised the manuscript for intellectual content. All authors reviewed and approved the final version of the manuscript.

10. **Conflict of interest**

The authors declare that the research was conducted in the absence of any commercial or financial relationships that could be construed as potential conflicts of interest.

11. **Publisher’s Note**

All claims expressed in this article are solely those of the authors and do not necessarily reflect the views of their affiliated institutions, publishers, editors, or reviewers. A product that may be evaluated or claimed by the manufacturer is not guaranteed or endorsed by the publisher.

**References:**

1.	Amin H, Swainson MG, Khan MA, Bukhari M: **Association of a Combined Body Mass Index and Regional Body Fat Percentage Metric With Fragility Fracture Risk: Evidence from a Large Observational Cohort**. *J CACHEXIA SARCOPENI* 2025, **16**(2):e13808.

2.	Luan A, Yao J: **An Update on Osteoporosis Screening: Advances, Applications, and the Role of Hand Surgeons and Allied Health Providers**. *J HAND SURG-AM* 2025.

3.	Libman H, Yu EW, Malabanan AO, Smetana GW: **How Would You Manage This Patient With Decreased Bone Density? Grand Rounds Discussion From Beth Israel Deaconess Medical Center**. *ANN INTERN MED* 2025, **178**(4):579-587.

4.	Reid IR, McClung MR: **Osteopenia: a key target for fracture prevention**. *LANCET DIABETES ENDO* 2024, **12**(11):856-864.

5.	Szybiak-Skora W, Cyna W, Lacka K: **New Insights in the Diagnostic Potential of Sex Hormone-Binding Globulin (SHBG)-Clinical Approach**. *BIOMEDICINES* 2025, **13**(5).

6.	Ji W, Pan B, Chen X, Lao Z, Yang W, Qian Y: **Mendelian randomization studies of risk and protective factors for osteoporosis: a systematic review and meta-analysis**. *FRONT ENDOCRINOL* 2024, **15**:1486188.

7.	Giannoulis MG, Martin FC, Nair KS, Umpleby AM, Sonksen P: **Hormone replacement therapy and physical function in healthy older men. Time to talk hormones?** *ENDOCR REV* 2012, **33**(3):314-377.

8.	Ensrud KE, Crandall CJ: **Osteoporosis**. *ANN INTERN MED* 2024, **177**(1):ITC1-ITC16.

9.	Qaseem A, Mount CA, Campos K, McLean RM, Tierney S, Cross JTJ, Fitterman N, Andrews RA, Basch P, Goldzweig CL *et al*: **Quality Indicators for Osteoporosis in Adults: A Review of Performance Measures by the American College of Physicians**. *ANN INTERN MED* 2023, **176**(10):1386-1391.

10.	Marriott RJ, Murray K, Adams RJ, Antonio L, Ballantyne CM, Bauer DC, Bhasin S, Biggs ML, Cawthon PM, Couper DJ *et al*: **Factors Associated With Circulating Sex Hormones in Men : Individual Participant Data Meta-analyses**. *ANN INTERN MED* 2023, **176**(9):1221-1234.

11.	Reid IR, McClung MR: **Osteopenia: a key target for fracture prevention**. *LANCET DIABETES ENDO* 2024, **12**(11):856-864.

12.	Simo R, Saez-Lopez C, Barbosa-Desongles A, Hernandez C, Selva DM: **Novel insights in SHBG regulation and clinical implications**. *TRENDS ENDOCRIN MET* 2015, **26**(7):376-383.

13.	Mun H, Liu B, Pham THA, Wu Q: **C-reactive protein and fracture risk: an updated systematic review and meta-analysis of cohort studies through the use of both frequentist and Bayesian approaches**. *OSTEOPOROSIS INT* 2021, **32**(3):425-435.

14.	van den Kieboom J, Bosch P, Plate JDJ, IJpma FFA, Kuehl R, McNally MA, Metsemakers W, Govaert GAM: **Diagnostic accuracy of serum inflammatory markers in late fracture-related infection: a systematic review and meta-analysis**. *BONE JOINT J* 2018, **100-B**(12):1542-1550.

15.	Larsson SC, Michaelsson K, Burgess S: **Mendelian randomization in the bone field**. *BONE* 2019, **126**:51-58.

16.	Cariou B, Charbonnel B, Staels B: **Thiazolidinediones and PPARgamma agonists: time for a reassessment**. *TRENDS ENDOCRIN MET* 2012, **23**(5):205-215.

17.	Lecka-Czernik B: **Diabetes, bone and glucose-lowering agents: basic biology**. *DIABETOLOGIA* 2017, **60**(7):1163-1169.

18.	Lecka-Czernik B, Rosen CJ: **Energy Excess, Glucose Utilization, and Skeletal Remodeling: New Insights**. *J BONE MINER RES* 2015, **30**(8):1356-1361.

19.	Manson JE, Crandall CJ, Rossouw JE, Chlebowski RT, Anderson GL, Stefanick ML, Aragaki AK, Cauley JA, Wells GL, LaCroix AZ *et al*: **The Women's Health Initiative Randomized Trials and Clinical Practice: A Review**. *JAMA-J AM MED ASSOC* 2024, **331**(20):1748-1760.

20.	Kahwati LC, LeBlanc E, Weber RP, Giger K, Clark R, Suvada K, Guisinger A, Viswanathan M: **Screening for Vitamin D Deficiency in Adults: Updated Evidence Report and Systematic Review for the US Preventive Services Task Force**. *JAMA-J AM MED ASSOC* 2021, **325**(14):1443-1463.

21.	Skrivankova VW, Richmond RC, Woolf BAR, Yarmolinsky J, Davies NM, Swanson SA, VanderWeele TJ, Higgins JPT, Timpson NJ, Dimou N *et al*: **Strengthening the Reporting of Observational Studies in Epidemiology Using Mendelian Randomization: The STROBE-MR Statement**. *JAMA-J AM MED ASSOC* 2021, **326**(16):1614-1621.

22.	Emdin CA, Khera AV, Kathiresan S: **Mendelian Randomization**. *JAMA-J AM MED ASSOC* 2017, **318**(19):1925-1926.

23.	Davey Smith G, Ebrahim S, Lewis S, Hansell AL, Palmer LJ, Burton PR: **Genetic epidemiology and public health: hope, hype, and future prospects**. *LANCET* 2005, **366**(9495):1484-1498.

24.	Li D, Zhou L, Cao Z, Wang J, Yang H, Lyu M, Zhang Y, Yang R, Wang J, Bian Y *et al*: **Associations of environmental factors with neurodegeneration: An exposome-wide Mendelian randomization investigation**. *AGEING RES REV* 2024, **95**:102254.

25.	Zhang B, He W, Pei Z, Guo Q, Wang J, Sun M, Yang X, Ariben J, Li S, Feng W *et al*: **Plasma proteins, circulating metabolites mediate causal inference studies on the effect of gut bacteria on the risk of osteoporosis development**. *AGEING RES REV* 2024, **101**:102479.

26.	Zhang B, He W, Pei Z, Guo Q, Wang J, Sun M, Yang X, Ariben J, Li S, Feng W *et al*: **Plasma proteins, circulating metabolites mediate causal inference studies on the effect of gut bacteria on the risk of osteoporosis development**. *AGEING RES REV* 2024, **101**:102479.

27.	Zhao J, Zeng X, Wang J, Liu L: **Association Between Calcium or Vitamin D Supplementation and Fracture Incidence in Community-Dwelling Older Adults: A Systematic Review and Meta-analysis**. *JAMA-J AM MED ASSOC* 2017, **318**(24):2466-2482.

28.	Mosekilde L, Vestergaard P, Rejnmark L: **The pathogenesis, treatment and prevention of osteoporosis in men**. *DRUGS* 2013, **73**(1):15-29.

29.	Bhasin S: **Testosterone supplementation for aging-associated sarcopenia**. *J GERONTOL A-BIOL* 2003, **58**(11):1002-1008.

30.	Schwartz AV, Backlund JC, de Boer IH, Rubin MR, Barnie A, Farrell K, Trapani VR, Gregory NS, Wallia A, Bebu I *et al*: **Risk factors for lower bone mineral density in older adults with type 1 diabetes: a cross-sectional study**. *LANCET DIABETES ENDO* 2022, **10**(7):509-518.

31.	Ikramuddin S, Billington CJ, Lee W, Bantle JP, Thomas AJ, Connett JE, Leslie DB, Inabnet WBR, Jeffery RW, Chong K *et al*: **Roux-en-Y gastric bypass for diabetes (the Diabetes Surgery Study): 2-year outcomes of a 5-year, randomised, controlled trial**. *LANCET DIABETES ENDO* 2015, **3**(6):413-422.

32.	Kahwati LC, LeBlanc E, Weber RP, Giger K, Clark R, Suvada K, Guisinger A, Viswanathan M: **Screening for Vitamin D Deficiency in Adults: Updated Evidence Report and Systematic Review for the US Preventive Services Task Force**. *JAMA-J AM MED ASSOC* 2021, **325**(14):1443-1463.

33.	Reid IR, Bolland MJ, Grey A: **Effects of vitamin D supplements on bone mineral density: a systematic review and meta-analysis**. *LANCET* 2014, **383**(9912):146-155.

34.	Bischoff-Ferrari HA, Willett WC, Wong JB, Giovannucci E, Dietrich T, Dawson-Hughes B: **Fracture prevention with vitamin D supplementation: a meta-analysis of randomized controlled trials**. *JAMA-J AM MED ASSOC* 2005, **293**(18):2257-2264.

35.	Vlasschaert C, Robinson-Cohen C, Chen J, Akwo E, Parker AC, Silver SA, Bhatraju PK, Poisner H, Cao S, Jiang M *et al*: **Clonal hematopoiesis of indeterminate potential is associated with acute kidney injury**. *NAT MED* 2024, **30**(3):810-817.

36.	Huang S, Liu S, Huang M, He J, Wang C, Wang T, Feng X, Kuang Y, Lu J, Gu Y *et al*: **The Born in Guangzhou Cohort Study enables generational genetic discoveries**. *NATURE* 2024, **626**(7999):565-573.

37.	Pairo-Castineira E, Rawlik K, Bretherick AD, Qi T, Wu Y, Nassiri I, McConkey GA, Zechner M, Klaric L, Griffiths F *et al*: **GWAS and meta-analysis identifies 49 genetic variants underlying critical COVID-19**. *NATURE* 2023, **617**(7962):764-768.

38.	Chen L, Zhernakova DV, Kurilshikov A, Andreu-Sanchez S, Wang D, Augustijn HE, Vich Vila A, Weersma RK, Medema MH, Netea MG *et al*: **Influence of the microbiome, diet and genetics on inter-individual variation in the human plasma metabolome**. *NAT MED* 2022, **28**(11):2333-2343.

39.	Yazar S, Alquicira-Hernandez J, Wing K, Senabouth A, Gordon MG, Andersen S, Lu Q, Rowson A, Taylor TRP, Clarke L *et al*: **Single-cell eQTL mapping identifies cell type-specific genetic control of autoimmune disease**. *SCIENCE* 2022, **376**(6589):eabf3041.

40.	Kousathanas A, Pairo-Castineira E, Rawlik K, Stuckey A, Odhams CA, Walker S, Russell CD, Malinauskas T, Wu Y, Millar J *et al*: **Whole-genome sequencing reveals host factors underlying critical COVID-19**. *NATURE* 2022, **607**(7917):97-103.

41.	Gaziano L, Giambartolomei C, Pereira AC, Gaulton A, Posner DC, Swanson SA, Ho Y, Iyengar SK, Kosik NM, Vujkovic M *et al*: **Actionable druggable genome-wide Mendelian randomization identifies repurposing opportunities for COVID-19**. *NAT MED* 2021, **27**(4):668-676.

42.	Traylor M, Persyn E, Tomppo L, Klasson S, Abedi V, Bakker MK, Torres N, Li L, Bell S, Rutten-Jacobs L *et al*: **Genetic basis of lacunar stroke: a pooled analysis of individual patient data and genome-wide association studies**. *LANCET NEUROL* 2021, **20**(5):351-361.

43.	Ginsburg O, Ashton-Prolla P, Cantor A, Mariosa D, Brennan P: **The role of genomics in global cancer prevention**. *NAT REV CLIN ONCOL* 2021, **18**(2):116-128.

44.	Firth J, Solmi M, Wootton RE, Vancampfort D, Schuch FB, Hoare E, Gilbody S, Torous J, Teasdale SB, Jackson SE *et al*: **A meta-review of "lifestyle psychiatry": the role of exercise, smoking, diet and sleep in the prevention and treatment of mental disorders**. *WORLD PSYCHIATRY* 2020, **19**(3):360-380.

45.	Ference BA, Ray KK, Nicholls SJ: **Mendelian Randomization Study of ACLY and Cardiovascular Disease. Reply**. *NEW ENGL J MED* 2020, **383**(7):e50.

46.	Sakaue S, Kanai M, Karjalainen J, Akiyama M, Kurki M, Matoba N, Takahashi A, Hirata M, Kubo M, Matsuda K *et al*: **Trans-biobank analysis with 676,000 individuals elucidates the association of polygenic risk scores of complex traits with human lifespan**. *NAT MED* 2020, **26**(4):542-548.

47.	Ray KK, Corral P, Morales E, Nicholls SJ: **Pharmacological lipid-modification therapies for prevention of ischaemic heart disease: current and future options**. *LANCET* 2019, **394**(10199):697-708.

48.	Tworoger SS, Zhang X, Eliassen AH, Qian J, Colditz GA, Willett WC, Rosner BA, Kraft P, Hankinson SE: **Inclusion of endogenous hormone levels in risk prediction models of postmenopausal breast cancer**. *J CLIN ONCOL* 2014, **32**(28):3111-3117.

49.	Friedenreich CM, Woolcott CG, McTiernan A, Ballard-Barbash R, Brant RF, Stanczyk FZ, Terry T, Boyd NF, Yaffe MJ, Irwin ML *et al*: **Alberta physical activity and breast cancer prevention trial: sex hormone changes in a year-long exercise intervention among postmenopausal women**. *J CLIN ONCOL* 2010, **28**(9):1458-1466.

50.	Tong R, Liang Z, Zhuo C, Bai X, Dao L, Yu L, Li L, Tong Z, Du Y, Xu L: **Causal associations between inflammatory bowel disease and sepsis: a two-sample Mendelian randomization study**. *BMC MED GENOMICS* 2025, **18**(1):74.

51.	Yoshiji S, Lu T, Butler-Laporte G, Carrasco-Zanini-Sanchez J, Su C, Chen Y, Liang K, Willett JDS, Wang S, Adra D *et al*: **Integrative proteogenomic analysis identifies COL6A3-derived endotrophin as a mediator of the effect of obesity on coronary artery disease**. *NAT GENET* 2025, **57**(2):345-357.

52.	Haglund A, Zuber V, Abouzeid M, Yang Y, Ko JH, Wiemann L, Otero-Jimenez M, Muhammed L, Feleke R, Nott A *et al*: **Cell state-dependent allelic effects and contextual Mendelian randomization analysis for human brain phenotypes**. *NAT GENET* 2025, **57**(2):358-368.

53.	Wang G, Wang J, Yang D, Liu L, Xu P: **Severe traumatic brain injury and risk for osteoporosis: a Mendelian randomization study**. *BMC MED GENOMICS* 2025, **18**(1):61.

54.	Zheng X, Li J, Ma Q, Gong J, Pan J: **Integrative analyses of mendelian randomization and bioinformatics reveal casual relationship and genetic links between COVID-19 and knee osteoarthritis**. *BMC MED GENOMICS* 2025, **18**(1):2.

55.	Wu J, Chen J, Huang M, Deng X, Deng J, Lau TY, Cao S, Ran H, Jiang Z, Luo J: **The causal effect of gut microbiota on hepatic encephalopathy: a mendelian randomization analysis**. *BMC MED GENOMICS* 2024, **17**(1):216.

56.	Guo W, Guo Y, Song S, Huang X, Zhang Y, Zhang A, Meng F, Chang M, Wang Z: **Causal effect of the age at first birth with depression: a mendelian randomization study**. *BMC MED GENOMICS* 2024, **17**(1):192.

57.	Henry A, Mo X, Finan C, Chaffin MD, Speed D, Issa H, Denaxas S, Ware JS, Zheng SL, Malarstig A *et al*: **Genome-wide association study meta-analysis provides insights into the etiology of heart failure and its subtypes**. *NAT GENET* 2025, **57**(4):815-828.

58.	Benson MD, Eisman AS, Tahir UA, Katz DH, Deng S, Ngo D, Robbins JM, Hofmann A, Shi X, Zheng S *et al*: **Protein-metabolite association studies identify novel proteomic determinants of metabolite levels in human plasma**. *CELL METAB* 2023, **35**(9):1646-1660.

59.	Chen J, Zhao J, Fu S: **Addressing sample overlap and meta-analysis imbalances in Mendelian randomization: a critical appraisal of protein targets for myocardial infarction**. *EUR HEART J* 2025.

60.	German J, Yang Z, Urbut S, Vartiainen P, Natarajan P, Pattorno E, Kutalik Z, Philippakis A, Ganna A: **Incorporating genetic data improves target trial emulations and informs the use of polygenic scores in randomized controlled trial design**. *NAT GENET* 2025.

Figure 1.Study Flow Chart：①Instrumental variables (IVs) must be strongly associated with the exposures (SHBG and candidate mediators).② IVs should not be associated with confounders of the exposure–outcome or mediator–outcome relationships.③ IVs influence the outcome (lumbar fracture) only through the exposures and mediators.Solid arrows denote validated causal or correlational paths, whereas dashed lines indicate the absence of correlation. Abbreviations: BMD, total body bone mineral density; SHBG, sex hormone–binding globulin; CRP, C-reactive protein; GHL, growth hormone levels; BTL, bioavailable testosterone levels; HbA1c, glycated hemoglobin; 25(OH)D, 25-hydroxyvitamin D.

Figure 2. Mendelian randomization estimates for the causal effect of SHBG on lumbar vertebral fracture risk. Forest plot of odds ratios (ORs) and 95% confidence intervals for a one–standard‐deviation increase in genetically predicted SHBG on fracture risk in FinnGen and UK Biobank, using IVW, MR‐Egger, weighted median, simple mode, and weighted mode methods.

Figure 3. Mendelian randomization estimates of the causal effect of SHBG on lumbar spine bone mineral density (BMD), overall and by age strata (0–15, 15–30, 30–45, 45–60, >60 years). Forest plot displays odds ratios (ORs) and 95% confidence intervals for a one–standard‐deviation increase in genetically predicted SHBG, as estimated by IVW (primary) alongside MR‐Egger, weighted median, simple mode, and weighted mode methods. Significant negative associations are observed in the 45–60 and >60 year groups, indicating that SHBG‐mediated reductions in BMD emerge in midlife and intensify in older age.

Figure 4. Causal effect estimates of lumbar spine bone mineral density (BMD) on lumbar vertebral fracture risk across the lifespan. Forest plot of inverse‐variance weighted odds ratios (ORs) and 95% confidence intervals for a one–standard‐deviation increase in BMD overall and by age strata (0–15, 15–30, 30–45, 45–60, >60 years). Significant protective effects are observed overall (OR = 0.673; 95% CI, 0.582–0.779; P = 1.04×10⁻⁷) and in the 0–15, 45–60, and >60 year groups, with non‐significant trends in the 15–30 and 30–45 year strata.

Figure 5. Causal effect estimates of SHBG on a panel of candidate mediators. Forest plot of inverse‐variance weighted odds ratios (ORs) and 95% confidence intervals for a one–standard‐deviation increase in genetically predicted SHBG on: whole‐body BMD (OR = 0.934; P = 0.0029), CRP (OR = 0.921; P = 0.0065), insulin medication use (OR = 0.9963; P = 6.1×10⁻⁶), growth hormone (OR = 1.081; P = 0.0133), bioavailable testosterone (OR = 0.758; P < 1×10⁻¹⁴⁵), HbA1c (OR = 0.859; P = 2.33×10⁻¹³), and 25‐hydroxyvitamin D (OR = 1.033; P = 0.0240).

Figure 6. Causal effects of candidate mediators on lumbar vertebral fracture risk. Forest plot of inverse‐variance weighted odds ratios (ORs) and 95% confidence intervals for a one–standard‐deviation increase in each mediator—lumbar spine BMD (overall and age‐stratified: 0–15, 15–30, 30–45, 45–60, >60 years), whole‐body BMD (GCST005348), C-reactive protein (CRP), insulin medication use, growth hormone (GH), bioavailable testosterone (Bio-T), HbA1c, and 25-hydroxyvitamin D (25(OH)D)—on vertebral fracture risk. Significant protective effects are observed for lumbar spine BMD overall (OR=0.673; 95% CI 0.582–0.779; P=1.04×10⁻⁷) and in the 0–15, 45–60, and >60 year groups, as well as for lower CRP (OR=0.856; 95% CI 0.738–0.993; P=0.039). Insulin use shows a positive association (OR≈22.3; P=0.0499), while other biomarkers exhibit non‐significant effects.

Figure 7. Mediation analysis of the SHBG to lumbar vertebral fracture pathway. Bar plot showing the proportion of the total effect (β_total) of SHBG on fracture mediated by (i) lumbar spine BMD (64.8%), (ii) C-reactive protein (63.8%), and (iii) insulin medication use (38.7%). Indirect effect estimates (β_indirect) with standard errors and corresponding odds ratios (OR_indirect) are annotated for each mediator.

Figure 8. Assessment of heterogeneity and pleiotropy across Mendelian randomization analyses. Bar plot of Cochran’s Q–derived P values for IVW heterogeneity alongside MR‐Egger intercept P values and MR‐PRESSO global test P values for each exposure–outcome pair. Significant heterogeneity (Q_IVW P < 0.05) is observed for most SHBG→mediator and mediator→fracture paths, while non‐significant MR‐Egger intercepts indicate minimal directional pleiotropy. MR‐PRESSO global tests detect outlier SNPs (P < 0.05) but correction does not alter causal estimates.

Supplementary Material Legends.

Table S1. Overview of the GWAS data in the current study

Table S2.The detailed information of SNPs chosen for instrumental variables of between SHBG and LF(EUR)

Table S3.The detailed information of SNPs chosen for instrumental variables of between SHBG and LF(UKB)

Table S4.The detailed information of SNPs chosen for instrumental variables of between SHBG and CRP

Table S5.The detailed information of SNPs chosen for instrumental variables of between SHBG and Insulin

Table S6.The detailed information of SNPs chosen for instrumental variables of between SHBG and GHL

Table S7.The detailed information of SNPs chosen for instrumental variables of between SHBG and BTL

Table S8.The detailed information of SNPs chosen for instrumental variables of between SHBG and HbA1c Levels

Table S9.The detailed information of SNPs chosen for instrumental variables of between SHBG and 25(OH)D Levels

Table S10.The detailed information of SNPs chosen for instrumental variables of between SHBG and BMD(0-15)

Table S11.The detailed information of SNPs chosen for instrumental variables of between SHBG and BMD(15-30)

Table S12.The detailed information of SNPs chosen for instrumental variables of between SHBG and BMD(30-45)

Table S13.The detailed information of SNPs chosen for instrumental variables of between SHBG and BMD(45-60)

Table S14.The detailed information of SNPs chosen for instrumental variables of between SHBG and BMD(>60)

Table S15.The detailed information of SNPs chosen for instrumental variables of between SHBG and BMD

Table S16.The detailed information of SNPs chosen for instrumental variables of between CRP and LF(EUR)

Table S17.The detailed information of SNPs chosen for instrumental variables of between Insulin and LF(EUR)

Table S18.The detailed information of SNPs chosen for instrumental variables of between GHL and LF(EUR)

Table S19.The detailed information of SNPs chosen for instrumental variables of between BTL and LF(EUR)

Table S20.The detailed information of SNPs chosen for instrumental variables of between HbA1c Levels and LF(EUR)

Table S21.The detailed information of SNPs chosen for instrumental variables of between 25(OH)D Levels and LF(EUR)

Table S22.The detailed information of SNPs chosen for instrumental variables of between BMD(0-15) and LF(EUR)

Table S23.The detailed information of SNPs chosen for instrumental variables of between BMD(15-30) and LF(EUR)

Table S24.The detailed information of SNPs chosen for instrumental variables of between BMD(30-45) and LF(EUR)

Table S25.The detailed information of SNPs chosen for instrumental variables of between BMD(45-60) and LF(EUR)

Table S26.The detailed information of SNPs chosen for instrumental variables of between BMD(>60) and LF (EUR)

Table S27.The detailed information of SNPs chosen for instrumental variables of between BMD and LF (EUR)
